# Unveiling *Lathyrus aphaca* L. as a Newly Identified Host for Begomovirus Infection: A Comprehensive Study

**DOI:** 10.3390/genes14061221

**Published:** 2023-06-03

**Authors:** Tehmina Bahar, Fasiha Qurashi, Muhammad Saleem Haider, Murad Ali Rahat, Fazal Akbar, Muhammad Israr, Ahmad Ali, Zahid Ullah, Fazal Ullah, Mohamed A. El-Sheikh, Ryan Casini, Hosam O. Elansary

**Affiliations:** 1 Department of Plant Pathology, Faculty of Agricultural Sciences, The University of Punjab, Lahore 54590, Pakistan; 2 Department of Forestry, Range & Wildlife Management, Faculty of Agriculture & Environment, The Islamia University of Bahawalpur, Bahawalpur 63100, Pakistan; 3 Department of Physiology, Biological Sciences, University of Veterinary and Animal Sciences, Lahore 54000, Pakistan; 4 Centre for Biotechnology and Microbiology, University of Swat, Swat 01923, Pakistanfazalakbar@uswat.edu.pk (F.A.); 5 Department of Forensic Sciences, University of Swat, Swat 01923, Pakistan; 6 Center for Plant Sciences and Biodiversity, University of Swat, Charbagh, Swat 01923, Pakistan; 7 State Key Laboratory of Grassland Agro-Ecosystems, College of Ecology, Lanzhou University, Lanzhou 730000, China; fazalbotanist@gmail.com; 8 Botany and Microbiology Department, College of Science, King Saud University, P.O. Box 2460, Riyadh 11451, Saudi Arabia; 9 School of Public Health, University of California, 2121 Berkeley Way, Berkeley, CA 94704, USA; 10 Plant Production Department, College of Food and Agriculture Sciences, King Saud University, P.O. Box 2460, Riyadh 11451, Saudi Arabia

**Keywords:** Geminiviridae, *Lathyrus aphaca*, monopartite begomovirus

## Abstract

The *Begomovirus* genus of the family *Geminiviridae* comprises the largest group of geminiviruses. Begomoviruses are transmitted by the whitefly complex (*Bemisia tabaci*) and infect dicotyledonous plants in tropical and subtropical regions. The list of begomoviruses is continuously increasing as a result of improvements in the methods for identification, especially from weed plants, which are considered a source of new viruses and reservoirs of economically important viruses but are often neglected during diversity studies. *Lathyrus aphaca* L. weed plants (yellow-flowered pea) with varicose veins and discoloration of the leaves were found. Amplified genomic DNA through rolling circular amplification was subjected to PCR analysis for the detection of the viral genome and associated DNA-satellites (alphasatellites and betasatellites). A full-length sequence (2.8 kb) of a monopartite begomovirus clone was determined; however, we could not find any associated DNA satellites. The amplified full-length clone of *Rose leaf curl virus* (RoLCuV) reserved all the characteristics and features of an Old World (OW) monopartite begomovirus. Furthermore, it is the first time it has been reported from a new weed host, yellow-flowered pea. Rolling circle amplification and polymerase chain reaction analysis of associated DNA satellites, alphasatellite, and betasatellite, were frequently accomplished but unable to amplify from the begomovirus-infected samples, indicating the presence of only monopartite Old World begomovirus. It is observed that RoLCuV has the capability to infect different hosts individually without the assistance of any DNA satellite component. Recombination in viruses is also a source of begomovirus infection in different hosts.

## 1. Introduction

*Geminivirus* is a broad genus of single-stranded circular DNA (ssDNA) viruses spread by a variety of insect vectors such as whiteflies, grasshoppers, treehoppers, and aphids and globally infecting many crops, vegetables, ornamental plants, and weeds all over the world, especially in tropical and subtropical areas. These Geminiviruses and their satellites have progressed through an evolutionary path of a coordinated network of protein interactions [1,2], as they have twinned icosahedral DNA particles with a dimension of 22 nm × 38 nm [3] in their genomic structure. Currently, geminiviruses are classified into fourteen distinct genera: *Begomovirus, Mastrevirus, Curtovirus, Becurtovirus, Eragrovirus, Turncurtovirus, Topocuvirus, Capulavirus, Grablovirus, Citlodavirus, Maldovirus, Mulcrilevirus, opunvirus,* and *Topilevirus* based on host range, structural organization, and insect vector type. [4,5]. 

The biggest group in the *Geminiviridae* family is the genus *Begomovirus*, which has over >400 ICTV-recognized virus species that can cause economically devastating illnesses to a variety of crops in tropical and subtropical locations worldwide [6]. Some of the identifying symptoms of begomovirus infection include vein yellowing, vein thickening, leaf curling, mosaic, leaf rolling, stunted plant development, and leaf enations [7,8]. Begomoviruses have two genomic components, DNA-A and DNA-B. Monopartite begomoviruses, also known as Old World (OW) begomoviruses, have a single DNA-A element, while bipartite begomoviruses, also known as New World (NW) begomoviruses, have both DNA-A and DNA-B components in their genome. Both components of the bipartite genome are essential for viral infections [9]. They are also associated with other satellite-like molecules, such as alphasatellites, deltasatellites, and betasatellites, which cause diseases in dicotyledonous plants worldwide [10]. Alphasatellites have highly conserved genome organization and are self-replicating but are dependent on a helper virus for encapsidation and transmission [11]. They consist of an origin of replication (Ori), a replication-associated protein of almost 36 kDa, and 200 nucleotides of an adenine-rich region [12]. Betasatellites encode a single gene, BetaC1, which plays an important role in the induction of symptoms and suppression of transcriptional and post-transcriptional gene silencing. They are clearly associated with helper components and share no sequence similarities with them except for a nona-nucleotide (TAATATT/AC) [13,14]. Deltasatellites are almost a quarter the size of begomoviruses, have non-coding DNA satellites, and are associated with bipartite New World begomoviruses [15]. Only a tiny quantity of monopartite begomoviruses, such as the Tomato yellow leaf curl Sardinia virus, may infect and cause sickness signs in plants that lack betasatellite (TYLCSV) [16]. Over time, natural changes occur in the genetic elements of these begomoviruses, leading to their evolution from plasmids to monopartite geminiviruses with satellites that infect monocots and dicots in the Old World (OW), which includes Europe, Africa, Asia, and Australia. In contrast, bipartite geminiviruses without satellites infect dicots in the New World (NW), which includes America [17]. The OW monopartite begomovirus DNA-A component is bigger (2.9 kb) than the NW bipartite begomovirus DNA-A component (approximately 2.6 kb). These begomoviruses also have an additional V_2_ ORF, which is not found in NW bipartite begomoviruses. However, previously identified two species of begomovirus, *Corchorus yellow vein virus* (CoYVV) and *Corchorus golden mosaic virus* (CoGMV), both found in the OW, have similar characteristics to NW begomoviruses that lack the V_2_ gene.

Members of the genus *Begomovirus* are mostly transmitted by the *B. tabaci. B. tabaci* infestations have wreaked havoc in tropical and subtropical areas across the world, producing significant disease epidemics and enormous crop output reductions. Due to the extensive presence of whiteflies, more crop species are likely to be infected by geminiviruses. As a result, the presence of whitefly-transmitted viruses in agriculture presents a significant problem for scientists attempting to maintain agricultural productivity and quality.

*L. aphaca* (yellow-flowered pea), with twining habitat peas, is an aggressive dicotyledonous weed and has certain characteristics for survival in a competitive environment [18]. It is a trailing annual broadleaf weed with characteristics of medium height [19,20]. It belongs to the family *Fabaceae* and severely infests wheat crops in irrigated and rain-fed areas. It is a highly noxious weed in the rice–wheat cropping system of Pakistan [21]. Its germination time remains from October to April, and before the wheat crop in early April, it drops seed before wheat harvesting and increases trouble in the next subsequent crop of the winter season [22]. It is an important forage crop that has gained attention due to its high protein content and ability to fix atmospheric nitrogen. It is also resistant to various biotic and abiotic stresses, making it an ideal crop for marginal lands [23]. It has been used traditionally in various medicinal formulations due to its anti-inflammatory, analgesic, and antioxidant properties. It has also been reported to possess anti-hyperglycemic and anti-diabetic activities, making it a promising candidate for the treatment of diabetes [24]. Nowadays, scientists are also considering it an important genetic resource due to its high genetic diversity, which makes it a valuable tool for breeding programs. Various genetic markers have been developed to study the genetic diversity and population structure of *L. aphaca* L. populations [25].

The discovery of a new virus is always an exciting development in the field of virology. In our study, we isolated the *Rose leaf curl virus* RoLCuV from a noxious weed, *L. aphaca,* for the first time. This virus belongs to the family *Geminiviridae,* genus *Begomovirus,* and is single-stranded ssDNA. The plant exhibited symptoms of varicose veins and discoloration of the leaves, which are characteristic of RoLCuV infection [26]. The discovery of RoLCuV in *L. aphaca* is significant because, prior to this study, there was no evidence to suggest that this virus could infect this plant species. This expands our knowledge about the host range of RoLCuV and the types of plants that can be affected by this virus. It is possible that other plants may also be susceptible to RoLCuV, and further research could shed light on this possibility. This discovery also highlights the importance of studying viruses and their diversity. It is fascinating to learn about the ability of viruses to infect a wide range of hosts and the mechanisms they use to do so. This knowledge is crucial for developing strategies to prevent the spread of viral infections and protect plants and crops from viral diseases. This finding expands our knowledge about the host range of RoLCuV and adds to our understanding of the diversity of viruses and their ability to infect a variety of hosts. Further research is needed to explore the implications of this discovery and determine the potential impact of RoLCuV on other plant species.

## 2. Materials and Methods

**Figure 1:**
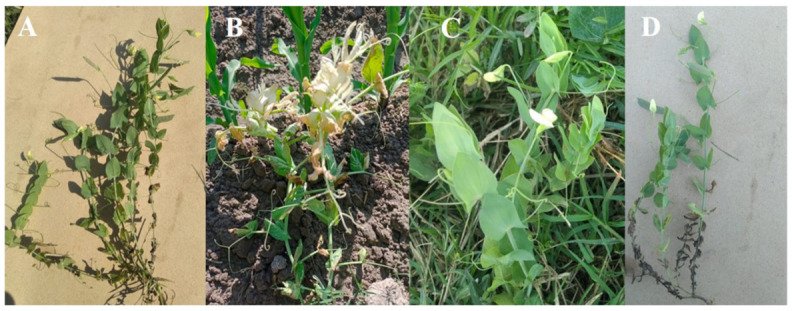
(**A**,**B**): *L. aphaca* (yellow-flowered pea) showing symptoms of varicose vein and discoloration collected from Jinnah Garden, Lahore, and Botanical garden of Punjab University, Quaid-e-Azam campus, Lahore, in 2018. (**C**,**D**); yellow-flowered pea showing symptoms of varicose veins collected from Jillani Park and Shalamar Garden, Lahore, respectively, in 2019.

Field survey for sample collection: Twelve (12) samples (33F1–33F12) of symptomatic plants were collected in 2018 and 2019, with three samples taken from each location: Jinnah Garden, Lahore (31.55° N, 74.33° E); Punjab University Botanical Garden, Quaid e Azam Campus, Lahore (31.29° N, 74.19° E); Jillani Park, Lahore (31.32° N, 74.20° E); and Shalamar Garden, Lahore (31.35° N, 74.22° E) and properly stored at −80 °C for the detection of viral symptoms and disease. These leaves are showing the symptoms of varicose veins and discoloration (Figure 1).

The DNA extraction and rolling circular amplification (RCA): By using the ionic detergent hexadecyltrimethylammonium bromide (CTAB) method, plant genomic DNA was extracted from infected leaves [27]. The technique of gel electrophoresis was used for the separation of DNA according to molecular size. The quantification of entire herbal genomic DNA was determined using a nano-drop (Denovix-DS11spectrophotometer), and the absorption of DNA quantification was resolute at a wavelength of 260 nm. For this, firstly, the machine was blanked with sterile distillate water (SDW) that has been cast off to break down DNA pellets to produce dilutions before the measurement of samples. For DNA measurement, whole plant genomic DNA (50 μg/mL) was used with an optical thickness of 1 at a 260 nm wavelength. To improve viral circular DNA, a rolling circular amplification mixture was made with DNA polymerase Phi29 (a 500 amplification kit of Illustra TempliPhi, GE*/ThermoScientific, Waltham, MA, USA). 100 ng of genomic DNA was combined with 2 μL of 10× phi29 reaction buffer of DNA polymerase, 1 μL of 50 μM randomly hexamer primers, 3 μL of 10 mM dNTPs, and remaining nuclease-free water to form an 18 μL total capacity mixture. RCA master tubes were then incubated for 3 min at 95 °C before being cooled on ice. 0.2 μL pyrophosphates, 0.75 μL phi29 deoxyribonucleic acid polymerase, and 1.05 μL nuclease-free H_2_O were further added to the RCA combination after it had cooled. The response combination was hatched for 20 h at 28 °C. The response was ended after incubation by suppressing the phi 29 DNA polymerase at 65 °C for 10 min. In PCR, the diluted RCA product was used to amplify the complete begomovirus complex [28]. 

Cloning of begomovirus genome: Initially, a coat protein polymerase chain reaction (PCR) assay was performed for the detection of high-fidelity viral genome products of RCA, which were carried out by degenerate primers AV Core (5′-GCCHATRTAYAGRAAGCCNAGRAT-3′) and AC Core (3′-GGRTTDGARGCATGHGTACANGCC-5′) [29], and the coat protein gene was amplified and confirmed. A pair of abutting primers, Begomo-AP-AF (5′GGCGACTGTGAAGAATGTTCATC-3′) and Begomo-AP-AR (5′-GTGCTGGGCTCATTATCAAACATG-3′), was used in PCR for the amplification of the full-length putative begomovirus genome and the amplified RCA product as a template. The machine was programmed for a preheating treatment of 94 °C for 5 min, followed by 35 cycles of 94 °C for 1 min, 52.5 °C for 1 min, and 72 °C for varying times (typically 1 min per 1000 nucleotides to be amplified), and a final incubation of 10 min at 72 °C. The ∼2.8 kbp DNA fragment was yielded on gel electrophoresis of the amplified begomovirus genome. After that, the PCR product was electrophoretically separated on a one percent agarose gel in tris-acetate EDTA (TAE) buffer. The required amplicons remained purified with a gel withdrawal kit (Illustra DNA/Gel PCR GFX Band Purification Kit) and cloned with path pTZ57R/T (Thermoscientific InsTAclone PCR replicating kit)/pGEM-T Easy Vector PCR replicating kit (Promega Cat. # A1360). Entire amount of ligation combination: (based on the duration of the DNA portion of 200 to 550 ng of PCR result), 2 μL 10× ligation buffer, 1 μL 5 sets T4 DNA Ligase, 1 μL 100 ng vector (pTZ57R/T), and sterile distilled water to create the final amount of 20 μL, hatched at 16 °C overnight. The ligation combination was heat-shock converted into proficient *Escherichia coli* cells and hatched at 37 °C for 60 min in a trembling incubator before being spread on hard LB average plates covering 100 uL of 24 mg/mL IPTG, 20 μL of 50 mg/mL X-Gal, and 100 ug/mL ampicillin and hatched for 16 h at 37 °C. In 5 mL of sterile liquid LB medium, white colonies were converted overnight in a shaker at 37 °C. *Apa1* and *Pst1* (Thermo Scientific kit with Cat.#ER0611 and ER1411, Waltham, MA, USA) were used for the digestion of plasmid DNA. M13F (5′-TGTAAAACGACGGCCAGT-3′) and M13R (5′-AGGAAACAGC TATGACCAT G-3′) universal primers were employed for the confirmatory colony PCR using a single colony as a template. To remove proteins, an equivalent amount of phenol and chloroform was combined through DNA solution, vortexed until milky, centrifuged at 14 thousand rpm for a time duration of 5 min, and the supernatant was moved into a fresh Eppendorf tube without disrupting the layer between the top aqueous phase and the phenol and chloroform. 1/10 quantity of 3 M NaCH₃COO (pH 5.2) and 2.5 quantities of icy absolute ethanol were added to the supernatant and stored at −80 °C for 24 h. The next day, the combination was centrifuged for an interval of 10 min at 14,000 rpm to pellet the deoxyribonucleic acid and then washed through 70% ethanol, air dried, and soaked in distilled H_2_O. First BASE Laboratory Sdn Bhd, Malaysia/ETON Biosciences, Inc., 5820 Oberlin Drive, Suite 108, San Diego, CA 92121 (http://www.etonbio.com/, accessed on 26 January 2021) in both directions were used for the sequence for the clone.

PCR amplification of alphasatellites and betasatellites: For amplification of alphasatellites and betasatellites polymerase chain reaction (PCR) assay was also performed for the detection of high-fidelity genome products of RCA by using DNA101(5′-CTGCAGATAATGATGTAGCTTACCAG-3′) and DNA102(5′-CTGCAGATCCTCCACGTGTATAG-3′) primers for alphasatellites and Beta01(5′-GGTACCACTACGCATCGCAGCAGCC-3′) and Beta02(5′-GGTACCTACCCTCCCAGGGGTACAC-3′) for betasatellites. Conditions for alphasatellite amplification were: initial denaturation at 95 °C for 5 min, and thereafter for 35 cycles, denaturation at 95 °C for 1 min; annealing at 50 °C for 1 min; extension at 72 °C for 1 min; and final extension at 72 °C for 10 min. For betasatellite amplification, cycling conditions were as described for betasatellites except that annealing was at 54.5 °C for 45 s.

Sequence analysis: correct and complete interpretation of the viral genome was done by manual scanning through a sequenced chromatogram. The open reading frame (ORF) finder at the NCBI website (http://www.ncbi.nlm.nih.gov/orffinder, accessed on 26 January 2021) was used to find out the protein-coding region of the full-length begomovirus genome in virion sense and complementary orientation. BLASTn analysis was used to find out the homologous sequence of the full-length begomovirus genome of the translated sequence present in Genbank, which is submitted for an accession number. Pairwise identity plots and color-coded distance matrices of begomovirus were identified by the sequence demarcation tool (SDT) with already submitted begomovirus at Genbank. The neighbor-joining (NJ) algorithm phylogenetic tree with 1000 bootstraps was constructed by using molecular evolutionary genetics analysis (MEGA7) software to find out the maximum likelihood of the obtained sequence. Seven different algorithms containing BootScan [30], SiScan [31], RDP [32], GENECONV [33], MaxChi [34] and Chimera [35] were used to determine recombination events of the obtained sequence by Recombination Detection Program (RDP4) [30]. The highest acceptable p-value (1 × 10^−5^) was used to predict the significant recombination breakpoint.

## 3. Results

### 3.1. Cloning and Sequencing of Begomovirus

**Figure 2:**
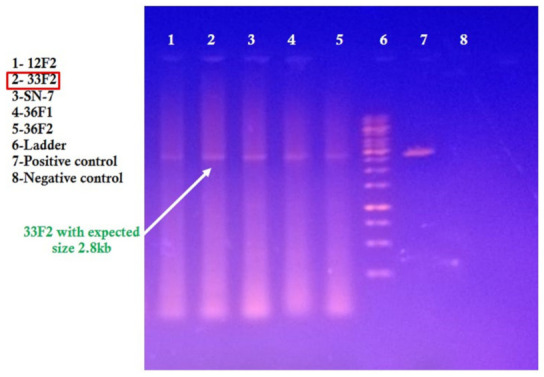
Gel well No. 2 reveals a prominent 2.8 kb DNA band corresponding to sample 33F2 by using Begomo-AP-AF & Begomo-AP-AR primers.

Initial analysis of the core coat protein sequences from all twelve symptomatic plant samples (33F1–33F12) revealed that three samples (33F1–33F3) collected from Punjab University Botanical Garden, Quaid e Azam Campus, Lahore, and one sample (33F4) collected from Jinnah Garden, Lahore, in 2018 were infected with a begomovirus. Additionally, one sample (33F9) collected from Jillani Park, Lahore, and one sample (33F11) collected from Shalamar Garden, Lahore, in 2019 also showed signs of begomovirus infection. Gel electrophoresis confirmed the presence of an approximately ±576bp-sized amplicon in all positive samples. The full-length clone of begomovirus was determined from only one sample (33F2) collected in 2018 from the Punjab University Botanical Garden, Quaid e Azam campus in Lahore. From sample 33F2, upon gel electrophoresis, a 2.8 kb band of polymerase chain reaction product was amplified by using abutting primers Begomo-AP-AF(5′-GGCGACTGTGAAGAATGTTCATC-3′) and Begomo-AP-AR (5′-GTGCTGGGCTCATTATCAAACATG-3′) and extracted DNA as a template (Figure 2). It consisted of 2756 nucleotides. Primers (DNA101;5′-CTGCAG ATAATGATGTAGCTTACCAG-3′, DNA102; 5′-CTGCAGATCCTCCACGTGTATAG-3′) and betasatellite (Beta01; 5′-ACGCGTATGGGCTGYCGAAGTTSAGACG-3′, Beta02; 5′-GGTACCACTACGCATCGCAGCAGCC-3′) were used to amplify the associated DNA satellite. A 2.8 kb band upon gel electrophoresis just confirmed the presence of monopartite begomovirus, while no associated satellite was amplified from the DNA of infected samples. The obtained PCR product was then cloned into the PGEM-T Easy vector (3015 bp) and again confirmed by restriction digestion with *ApaI* and *PstI* enzymes. After confirmation, the clone was sent for sequencing, and upon obtaining the partial sequences, full sequencing was done in its entirety. Bioinformatics analysis of clone 33F2 was performed for sequence comparison, organization of the genome, SDT (Sequence Demarcation Tool), pedigree analysis (construction of the phylogeny tree), and to observe the pattern of recombination. 

### 3.2. Genome Organization and Sequence Demarcation Tool Analysis of Clone-33F2; RoLCuV 

The clone 33F2 was analyzed by the NCBI BLAST (https://blast.ncbi.nlm.nih.gov.Blast.cgi, accessed on 26 January 2021) database with homology to the desired sequences, which are already submitted in GenBank. The old world (DNA-A) begomovirus was isolated from yellow-flowered pea (*L. aphaca*) hosts exhibiting begomovirus-like symptoms of leaf rolling and upward cup-shape curling. The obtained sequences of clone 33F2 were of the *Rose leaf curl virus* RoLCuV, submitted to GenBank with accession number MW528414. The genome size of the RoLCuV *Rose leaf curl virus* consisted of 2756 bp, showing all characteristic features of typical monopartite OW begomoviruses (V2, CP, Rep, TrAp, Ren, and C4) previously reported (Table 1). RoLCuV *Rose leaf curl virus* was showing the highest similarity (98.1%) with MN746285; RoLCuV was earlier reported from *P. granatum*, according to the Sequence Demarcation Tool (SDT) analysis mentioned in Table 2.

### 3.3. Phylogenetic Analysis of Clone-33F2; RoLCuV; MW528414

**Figure 3:**
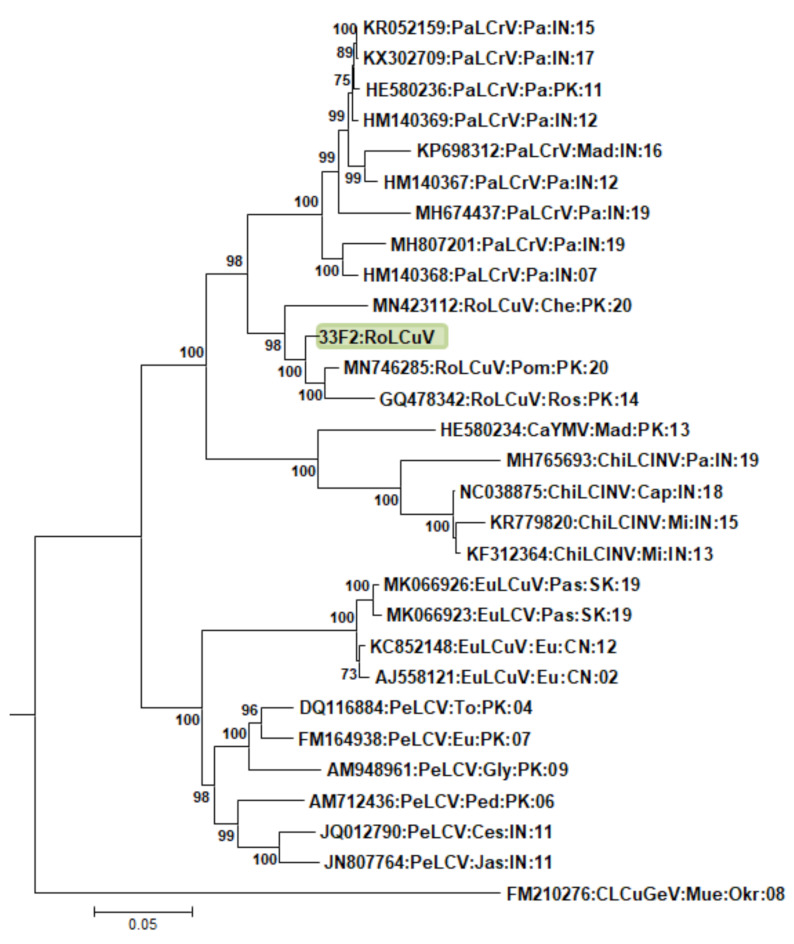
Phylogenetic dendrograms based on complete nucleotide sequences of begomovirus using Neighbor-Joining (NJ) algorithm in MEGA7. Isolate identified from Pakistan showed green background. Horizontal lines represent nucleotide substitutions per site. Numeric values at branch nodes represent percent bootstrap values higher than 60 (1000 replicates). All isolates used for comparison represented by their respective accession numbers in the tree. All abbreviations for begomovirus isolates follow the guidelines outlined in a previous publication. [36]. All abbreviations for begomovirus isolates are according to Brown et al. (2015) [37].

MEGA-7 software was used to build a phylogenetic tree of RoLCuV; MW528414. RoLCuV are also reported from different hosts, including pomegranate (*P. granatum*), Chinese rose (*H. rosasinensis*), wild spinach (*C. album*) in Pakistan, and rose (*R. indica*) from India, but this is the first time they have been reported from a new host, yellow-flowered pea (Figure 3).

### 3.4. Recombination Analysis of 33F2; RoLCuV; MW528414

In the present study, a putative recombination breakpoint was observed by using the recombination detection program (RDP) analysis in *Rose leaf curl viruses* 33F2; RoLCuV; MW528414. A recombination event was noticed at nucleotide coordinate R1; −1487–145, with the major parent *Catharanthus yellow mosaic virus* (HE580234; CaYMV) showing 29.8% similarity, while the minor parent devouring showed similarity with *Euphorbia leaf curl virus* (KC852148; EuLCV) mentioned in Table 3.

## 4. Discussion

Geminiviruses are a group of rapidly emerging plant viruses that cause considerable damage to cultivated plants around the world. In addition to cultivated plants, many species of non-cultivated plants, such as weeds and ornamental plants, are important hosts of geminiviruses and, therefore, may serve as virus reservoirs for field crop plants. Furthermore, ornamental plants and weeds may act both as ‘alternate hosts’ as well as ‘mixing vessels’ that yield new viruses/virus strains by recombination and component exchange due to their frequent harboring of multiple viruses. There are many research findings that clearly predict that begmoviruses infecting a wide range of cultivated crop plants, ornamental plants, vegetables, and weeds may have evolved through recombination in the weed hosts. The analysis of coat protein (CP, AV2/V2) genes demonstrates their pivotal roles in various biological processes. These genes are intricately associated with CP expression, overcoming host defenses, triggering responses to double-stranded RNA, facilitating movement, determining symptom manifestation, and regulating gene silencing. In this study, the amplified full-length sequence of the *Rose leaf curl virus* RoLCuV reserved all characteristics and features of the old-world monopartite begomovirus. *Rose leaf curl virus* RoLCuV and associated betasatellites were also earlier reported from ornamental hosts: RoLCuV (GQ478342) with associated *Digera arvensisn yellow vein betasatellite* (DiAYVB; GQ478344) in Pakistan [38], from *Rosa chinensis,* and also from *R. indica* associated with *Digera leaf curl betasatellite* (DiAYVB; KF584009) in India [26]. Only a single component of DNA-A without association with any DNA satellites was also reported from the woody host pomegranate (*P. granatum*, MN746285) in Pakistan [37]. However, it is the first time it has been reported from a new weed host, yellow-flowered pea (*L. aphaca*). RCA and PCR analysis of associated DNA satellites, alphasatellite and betasatellite, were frequently accomplished but unable to amplify from the begomovirus-infected samples, indicating the presence of only monopartite Old World begomovirus. It was also earlier reported from pomegranates.

Recently, RoLCuV has been identified in a new host, the yellow-flowered pea (*L. aphaca*). RCA and PCR analyses were conducted to detect the presence of associated DNA satellites, but only the monopartite old-world begomovirus was detected. This suggests that RoLCuV may be evolving and adapting to new hosts, which could have implications for crops. Recombination events are known to contribute to the emergence and evolution of viruses, and a putative recombination breakpoint was observed in RoLCuV. This may be the main reason for the upsurge of begomovirus infections in different hosts. The identification of RoLCuV in a new host emphasizes the importance of monitoring the emergence and evolution of viruses in different hosts to prevent the spread of diseases. The detection and characterization of RoLCuV and associated DNA satellites are important for developing effective control strategies. Begomoviruses are transmitted by whiteflies, which are difficult to control. Therefore, the use of resistant varieties, cultural practices, and integrated pest management strategies may be effective in managing the spread of begomoviruses.

Finally, the identification of RoLCuV in a new host, *L. aphaca*, highlights the potential for the emergence and evolution of begomoviruses in different hosts. The detection and characterization of RoLCuV and associated DNA satellites are important for developing effective control strategies to prevent the spread of diseases. The presence of a putative recombination breakpoint in RoLCuV suggests that recombination events may be contributing to the evolution of this virus. Therefore, continued monitoring of the emergence and evolution of begomoviruses in different hosts is essential for the development of effective management strategies.

## 5. Conclusions

Overall, the discovery of RoLCuV in *L. aphaca* is a new and exciting development in the field of virology. This study has shed light on the prevalence of begomoviruses in global agriculture, highlighting the need for timely monitoring and control of their spread. The study also revealed the isolation of the Rose leaf curl virus (RoLCuV) from *L. aphaca*, a noxious weed that severely infests wheat crops in Pakistan.

It was also found that RoLCuV, a characteristic Old World begomovirus, was not associated with DNA satellites and had the ability to infect different hosts individually without the assistance of any DNA satellite component. This is an important finding, as it suggests that RoLCuV may have a wider host range than previously thought and highlights the importance of early detection and control of geminiviruses to prevent their spread to important crops. The isolation of RoLCuV from *L. aphaca*, a noxious weed that severely infests wheat crops in Pakistan, is particularly concerning. This weed is difficult to control, and its presence can significantly reduce wheat yields, which are already low in the region due to a range of environmental and socio-economic factors. The fact that RoLCuV has been found in this weed suggests that it may be able to infect other important crops in the region and highlights the need for effective control measures to be implemented as soon as possible. 

One of the key findings of the study was that recombination in viruses is a source of begomovirus infection in different hosts. The study found evidence of recombination events in begomoviruses from different hosts, suggesting that this process plays an important role in the evolution and spread of these viruses. This is an important finding, as it suggests that efforts to control begomoviruses must take into account the potential for these viruses to rapidly evolve and adapt to new hosts and environments. These findings will be valuable for researchers who are studying the diversity and evolution of viruses, as well as for those who are working to develop strategies to control the spread of plant viruses. By implementing effective control measures and continuing to study these viruses, we can work towards reducing the impact of begomovirus infections in agriculture and ensuring food security for future generations.

## Figures and Tables

**Table 1:** Predicted Open Reading Frames (ORFs) of clone-33F2; RoLCuV.

ORFs	Position (nt Coordinates)	Size of ORFs (nt)	Predicted Protein Size (Amino Acids)
Pre-Coat Protein (V2)	144–509	366	121
Coat Protein (CP)	304–1074	771	256
Replication Associated Protein (Rep)	2605–1547	1059	352
Transcription Activator Protein (TrAp)	1623–1216	408	135
Replication Enhancer Protein (Ren)	1475–1071	405	134
C4	2454–2152	303	100

**Table 2:** The Sequence Demarcation Tool (SDT) analysis was done for begomovirus (DNA-A) clone-33F2; RoLCuV based on percent nucleotide identity; here, a color-coded matrix of pairwise similarity scores confirmed the clone; RoLCuV-33F2; MW528414 is fitted in *Rose leaf curl viruses*; RoLCuV.

Accession No.	Acronyms	Host Species	Country	Year	Length (bp)	% Identity
MN746285	RoLCuV	*P. granatum*	Pakistan	2020	2756	98.1
GQ478342	RoLCuV	*Hibiscus rosa-sinensis*	Pakistan	2014	2780	95.8
MN423112	RoLCuV	*Chenopodium album*	Pakistan	2020	2762	94.5
HM140368	PaLCrV	*Carica papaya*	India	2007	2736	92.6
HE580236	PaLCrV	*C. papaya*	Pakistan	2013	2737	91.9
KR052159	PaLCrV	*C. papaya*	India	2015	2736	91.8
KX302709	PaLCrV	*C. papaya*	India	2017	2736	91.8
HM140369	PaLCrV	*C. papaya*	India	2012	2736	91.7
MH807201	PaLCrV	*C. papaya*	India	2019	2736	91
HM140367	PaLCrV	*C. papaya*	India	2012	2736	90.6
MH674437	PaLCrV	*C. papaya*	India	2019	2738	89.5
KP698312	PaLCrV	*Catharanthus roseus*	India	2016	2736	89.4
FM164938	PeLCV	*Euphorbia pulcherrima*	Pakistan	2007	2759	85.2
HE580234	CaYMV	*C. roseus*	Pakistan	2013	2754	84.6
DQ116884	PeLCV	*Solanum lycopersicum*	Pakistan	2004	2759	
AM712436	PeLCV	*Pedilanthus tithymaloides*	Pakistan	2006	2764	84.2
JQ012790	PeLCV	*Cestrum nocturnum*	India	2011	2764	84.1
JN807764	PeLCV	*Tabernaemontana coronaria*	India	2011	2764	83.8
AM948961	PeLCV	*Glycine max*	Pakistan	2009	2760	83.6
NC038875	ChiLCINV	*Capsicum annuum*	India	2018	2755	83.5
KF312364	ChiLCINV	*Mentha spicata*	India	2013	2749	
MK066923	EuLCV	*Passiflora edulis*	South Korea	2019	2745	83.3
KC852148	EuLCuV	*E. pulcherrima*	China	2012	2745	83.2
AJ558121	EuLCuV	*E. pulcherrima*	China	2002	2746	83.1
MK066926	EuLCuV	*P. edulis*	South Korea	2019	2745	82.8
MH765693	ChiLCINV	*C. papaya*	India	2019	2755	82.7
KR779820	ChiLCINV	*Mentha arvensis*	India	2015	2749	82.4

**Table 3:** One recombination breakpoint was observed in 33F2-RoLCuV; MW528414.

Recombination Event	Beginning Breakpoint	Ending Breakpoint	Minor Parent	Major Parent	Probability Score
R1	145	1487	Unkonwn (KC852148;EuLCV)	HE580234; CaYMV (29.8%)	5.746 × 10^−92^–1412 × 10^−64^

## Data Availability

All data are available within this publication.
